# Correlation between the Expression of Angiogenic Factors and Stem Cell Markers in Human Uveal Melanoma

**DOI:** 10.3390/life10120310

**Published:** 2020-11-25

**Authors:** Klára Fodor, Éva Sipos, Nikoletta Dobos, János Nagy, Zita Steiber, Gábor Méhes, Kata Dull, Lóránt Székvölgyi, Andrew V. Schally, Gábor Halmos

**Affiliations:** 1 Department of Biopharmacy, Faculty of Pharmacy, University of Debrecen, 4032 Debrecen, Hungary; fodor.klara@pharm.unideb.hu (K.F.); sipos.eva@pharm.unideb.hu (É.S.); dobos.nikoletta@pharm.unideb.hu (N.D.); 2 Clinical Center, Department of Oncology, University of Debrecen, 4032 Debrecen, Hungary; nagyjanos@unideb.hu; 3 Clinical Center, Department of Ophthalmology, University of Debrecen, 4032 Debrecen, Hungary; steiber.zita@med.unideb.hu; 4 Department of Pathology, Faculty of Medicine, University of Debrecen, 4032 Debrecen, Hungary; gabor.mehes@med.unideb.hu (G.M.); kata.dull@med.unideb.hu (K.D.); 5 MTA-DE Momentum, Genome Architecture and Recombination Research Group, Department of Biochemistry and Molecular Biology, Faculty of Medicine, University of Debrecen, 4032 Debrecen, Hungary; lorantsz@med.unideb.hu; 6 Veterans Affairs Medical Center, Endocrine, Polypeptide and Cancer Institute, Miami, FL 33101, USA; Andrew.Schally@va.gov; 7 Sylvester Comprehensive Cancer Center, Department of Medicine, Department of Pathology, Divisions of Hematology Oncology and Endocrinology, Miller School of Medicine, University of Miami, Miami, FL 33101, USA

**Keywords:** uveal melanoma, cancer stem cell, angiogenesis, FZD6, VEGFA

## Abstract

Uveal melanoma (UM) is the most common malignant tumor of the eye with extremely high metastatic potential. UM tumor cells can disseminate only hematogenously, thus, angiogenic signals have a particular role in the prognosis of the disease. Although the presence of cancer stem cells (CSCs) in densely vascularized UMs has been reported previously, their role in the process of hematogenous spread of UM has not been studied. In this study, we investigated the regulation of angiogenesis in UM in correlation with the presence of CSCs. Seventy UM samples were collected to analyze the expression of CSC markers and angiogenic factors. The expression of CSC markers was studied by RT-PCR, Western blotting techniques and IHC-TMA technique. RT-PCR showed high expression of CSC markers, particularly nestin, FZD6 and SOX10 and somewhat lower expression of NGFR. The protein expression of FZD6, HIF-1α and VEGFA was further evaluated in 52 UM samples by the IHC-TMA technique. We report here for the first time a significant correlation between FZD6 and VEGFA expression in UM samples. The observed correlation between FZD6 and VEGFA suggests the presence of CSCs in UM that are associated with the vascularization process.

## 1. Introduction

Although uveal melanoma (UM) is a very rare disease, it is the most common primary intraocular malignancy in adults. Ultimately, up to 50% of the patients with primary uveal melanoma develop distant metastasis, the liver being involved in up to 90% of patients. The median survival of the patients with metastasis has been reported to be 4–5 months [1,2,3,4]. Consequently, for many years the major focus of research has been the identification of prognostic factors associated with the tumor progression and attempts to overcome the resistance of the tumor against combined targeted therapies [5,6,7,8]. Recent studies have shown that angiogenic factors, e.g., vascular endothelial growth factors (VEGF) or hypoxia-inducible factor 1-alpha (HIF1A) are overexpressed not only in tissue specimens, but also in UM cell lines under normoxic and hypoxic conditions [9,10,11,12]. It is well documented that VEGF and HIF1A are the key mediators of vasculogenesis, angiogenic remodeling and angiogenic sprouting of aggressive tumors as well, e.g., UM [6,8,10,13]. Because of the pathology of the eye, UM tumor cells can disseminate only hematogenously, thus, angiogenic signals have a particular role in the prognosis of the disease [6,8]. Although the correlation between the expression of VEGFA and tumor size or the development of distant metastasis is frequently analyzed in patients with UM, the exact function of VEGFA is still not established [7,14,15]. VEGF expression can be driven by many factors that are characteristic features for tumors, including oncogene expression, e.g., ras, src, erbB2/human epidermal growth factor receptor 2 (HER2), EGFR and also hypoxia [7]. Therefore, targeting transcriptional regulators may represent a promising therapeutic option in UM or conversely, these factors might contribute to the development of resistance against anti-VEGF targeted therapies. This can be the explanation for the studies demonstrating controversial or variable results in cell lines and animal models. El Filali et al. have demonstrated an acceleration of tumor growth in mouse models injected with anti-angiogenic drug bevacizumab; although, previous in vitro tests suggested a reduction in the proliferation of bevacizumab treated cells under hypoxic conditions [16]. Yang et al. reported a successful reduction of the tumor volume of UM and the number of distant metastases in mouse models treated by intraperitoneally administered bevacizumab [17]. In another clinical study, all patients received intraocular treatment with ranibizumab, a molecule that was developed from bevacizumab for the treatment of age-related macular degeneration. All patients showed tumor progression, therefore, this study had to be terminated and ranibizumab was suggested only as an adjunct treatment [18].

The use of anti-angiogenic therapy as a primary treatment has been reported to show limited efficacy or even the acceleration of tumor growth in many other clinical studies [19,20,21,22].

In order to overcome the potential resistance of UM tumors against anti-angiogenic therapies and to improve their efficacy, many other studies have investigated the combined administration of anti-VEGF drugs with other targeted or conventional anticancer drugs [5,6,23,24]. According to Tabernero and Ellis et al., the VEGF signaling pathway is upregulated by EGFR expression and conversely, VEGF upregulation independent of EGFR signaling seems to contribute to resistance to therapies applying EGFR inhibitors [7,25,26]. Based on these relationships, resistance to EGFR inhibition is common, correlating with increased VEGF expression and poor outcomes. Studies investigating the molecular background of failures of the anti-VEGF therapies show upregulation of the key regulators of angiogenesis in tumors. Thus, these changes may promote tumorigenesis and metastatic dissemination in a long run [12,16,27].

Taken together, therapeutic approaches to anti-angiogenic therapies are controversial.

Targeting tumor neoangiogenesis may only be successful if the effect of the tumor microenvironment is considered. Recent studies have demonstrated that VEGF binds primarily to its receptors on endothelial cells but may also act on hematopoietic or neural stem cells [14,28]. In addition, a primitive neural or ectodermal cell stem cell-like profile with significantly higher nestin gene expression was observed in densely vascularized UM specimens with a greater risk of metastasis [29,30,31]. Moreover, it has also been confirmed that UM tumor cells may enter the bloodstream, however, based on the cancer stem cell-hypothesis, this process is probably supported by CSCs [32,33,34,35,36]. To date, there are only limited studies examining the presence of CSCs in UM [29,30,34,37].

There is mounting evidence for the existence of CSCs in many forms of cancer, apparently, these cells could be responsible for increased resistance against a variety of anti-tumor treatment modalities and for tumor dissemination [33,38,39]. Like all stem cells, they can undergo asymmetric cell division and renew themselves producing cells with heterogenous phenotypes with chemoresistance. 

There is clear evidence for the complex process of neoangiogenic signals, the failures of anti-VEGFA treatments in UMs, the binding affinity of VEGF to hematopoietic or neural stem cells, and the presence of CSCs in circulating or metastatic UM tumors. One could still ask whether targeting the angiogenic factors is the real key to modifying the tumorigenesis of UM or are these processes only the consequences of other unknown key oncogenic factors like CSCs?

Trying to answer this question, in the present study we aimed to evaluate the expression of cancer stem cell markers and angiogenic factors in UM and to understand the relationships between the expression of the detected stem cell markers and vasculogenic mimicry patterns in primary UM tumors.

In order to assess the presence of CSCs in UM samples, we investigated the expression of five melanocyte stem cell markers by RT-PCR (reverse transcription- polimerase chain reaction), Western blotting and immunohistochemistry (IHC) using the tissue microarray technique (IHC-TMA).

We hope that demonstrating statistical relationship and relative prognostic significance among the expression of CSC factors in comparison with the survival of UM patients can provide new insights into the pathogenesis of UM. CSCs might serve as potential new biomarkers for the regulation of angiogenesis in UM. Since the therapy for this highly metastatic and chemoresistant tumor is not adequate, our work may be of assistance to identify a novel relationship among various molecular targets for the prevention of metastases of UM.

## 2. Materials and Methods

### 2.1. Patients and Tumor Samples

The local Institutional Ethics Committee approved the collection and use of the specimens for the current study and informed consent was obtained from the patients (ID number: DERKEB/IKEB 4172-2014). Primary human uveal melanoma (UM) specimens were obtained from 70 patients, aged between 30–84 years at the time of enucleation, at the Department of Ophthalmology, Clinical Center, University of Debrecen, Hungary. After surgical removal, 52 tissue specimens were paraffin-embedded, 18 specimens were snap-frozen and stored at −70 °C until isolation of RNA and protein. Conventional histopathological examination was performed on all tumors by investigation of S100 and HMB45 melanoma markers. In addition, the origin of the tumor was confirmed by the expert opinion of two independent pathologists.

Clinical features were recorded for each patient, including age, sex, tumor cell type, thickness and diameter of the tumor sample, sclera and nervus opticus involvement, previous ruthenium applicator therapy and the final status of the patient. A limitation of this study was that it included only primary UMs treated by enucleation.

### 2.2. RNA Isolation and Reverse Transcription-PCR of Cancer Stem Cell Markers

Total RNA was isolated from 18 primary UM tissue specimens using AllPrep DNA/RNA/Protein Mini kit (Qiagen, Hilden, Germany) according to the manufacturer’s instructions. Amounts of 250 ng RNA from each sample were reverse transcribed into cDNA by using the QuantiTect Reverse Transcription kit (Qiagen, Hilden, Germany) in a final volume of 20 μL. The expression of stem cell markers was studied by RT-PCR using gene specific primers for frizzled class receptor 6 (FZD6), NESTIN, nerve growth factor receptor (NGFR), prominin 1 (PROM1) and SRY-box transcription fator 10 (SOX10) genes (Sigma-Aldrich Corporation, St. Louis, MO, USA) (Table 1). As an internal control, β-actin housekeeping gene was used. Primers were designed using Primer3web software. One μL of cDNA was amplified in a 25 μL solution containing 1.5 mM MgCl_2_, 1x PCR buffer (Fermentas, Vilnius, Lithuania), 0.3 mM of each deoxynucleotide (Promega, Madison, WI, USA), 1 unit of DNA polymerase (Fermentas, Vilnius, Lithuania) and 0.25 μM of each primer. Samples were subjected to 35 cycles of 95 °C for 45 s, 60 °C for 30 s, then 72 °C for 1.5 min with a final extension of 10 min at 72 °C. PCR cycles were performed using the Bio-Rad C1000^TM^ Thermal Cycler (Bio-Rad Laboratories, Hercules, CA, USA) instruments. An amount of 10 μL of each amplification reaction was then electrophoretically separated on 1.5% agarose gel, stained with GelRed (Biotinum, Haywood, CA, USA), and visualized under UV light.

### 2.3. SDS-PAGE and Western Blot Analysis of FZD6, HIF-1α and VEGFA Protein Expression

Total protein was isolated from 18 primary UM tissue specimens using AllPrep DNA/RNA/Protein Mini kit according to the manufacturer’s instructions (Qiagen, Hilden, Germany). The final pellet was resuspended in 100 µL of protein dissolving buffer and stored until use at −80 °C. After measurement of protein concentration, 40 μg protein per sample was separated on 12% Sodium Dodecyl Sulphate Polyacrylamide Gel Electrophoresis (SDS PAGE) and transferred to polyvinylidene fluoride (PVDF) membranes, incubated for 2 h at room temperature in Tris buffered saline containing 0.1% Tween-20 and 5% non-fat dry milk, followed by incubation with a goat polyclonal full-length frizzled-6 receptor (FZD6) antibody (E-19; sc-32148) in 1:100 (Santa Cruz Biotechnology, Dallas, TX, USA); HIF1A (HIF1A (H-206); sc-10790, Santa Cruz Biotechnology, Dallas, TX, USA) rabbit polyclonal antibody in 1:200; VEGFA (VEGF (A-20; sc-152, Santa Cruz Biotechnology, Dallas, TX, USA) rabbit polyclonal antibody in 1:100 and Glyceraldehyde-3-Phosphate Dehydrogenase (GAPDH) (D16H11 XP^®^, Cell Signaling Technology, Danvers, MA, USA) rabbit monoclonal antibody in 1:1000 at 4 °C overnight. Primary antibodies were labeled by alkaline phosphatase conjugated secondary antibodies (Santa Cruz Biotechnology, Dallas, TX, USA) and detected using AP Conjugate Substrate Kit (Bio-Rad Laboratories, Hercules, CA, USA). Results were acquired and analyzed with the Molecular Image Chemidoc XRS+ System using Image Lab Software 5.2 (Bio-Rad Laboratories, Hercules, CA, USA).

### 2.4. Tissue Microarray Construction

TMA blocks were created by a computer-controlled TMA Master instrument (3DHISTECH, Budapest, Hungary). Briefly, the recipient paraffin block with 82 holes arranged in 7 columns and 12 rows was formed by 52 UMs, besides 12 livers and 2 normal nevi as positive and negative controls. Cores of 1.0 mm from the donor paraffin blocks were punched out with the Paraffin-Punch-Extractor and were arrayed in the recipient paraffin block. Each sample was represented at least in duplicate to avoid inadequate sampling.

### 2.5. Histopathology and Immunohistochemistry

Formalin-fixed paraffin-embedded, 3–4 μm thick tissue samples from enucleation were obtained, deparaffinized, rehydrated using ethanol, and treated with EnVision™ FLEX Peroxidase-Blocking Reagent (DM821, Dako, Glostrup, Denmark) to inhibit endogenous peroxidase activity. Heat-induced epitope retrieval was performed in Tris/EDTA-based buffer (pH: 9.0) (Novocastra™ Epitope Retrieval Solution pH9, RE7119) (Leica Microsystems, Wetzlar, Germany). Blocking step was performed using 2.5% normal goat or rabbit serum (Millipore Sigma, Burlington, MA, USA). After rinsing 3 times in EnVision™ FLEX Wash Buffer (pH:7.6, 5 min each, (DM831, Dako, Glostrup, Denmark), sections were incubated in monoclonal antibody specific for VEGF (A-20; sc-152) in 1:100 (Santa Cruz Biotechnology, Dallas, TX, USA); for frizzled-6 (FZD6 (E-19; sc-32148) in 1:100 (Santa Cruz Biotechnology, Dallas, TX, USA) and for HIF-1A (Pab50130, Covalab, Villeurbanne, France) in 1:3000 at room temperature in wet chamber at 4 °C overnight. After rinsing 3 times in EnVision™ FLEX Wash Buffer (pH: 7.6, 5 min each), sections were treated with anti-goat IgG (Fab)2 linked to horseradish-peroxidase (HRP) of Impress^TM^ HRP polymer detection kit (MP7405, Vector Labs, Peterborough, UK) or with anti-rabbit IgG (Fab)2 linked to horseradish-peroxidase (HRP) of EnVision™ FLEX/HRP (DM822, Dako, Glostrup, Denmark). Signals were developed using VectorVIP DAB+ Chromogen Substrate Kit (SK-4600, Vector^®^ Labs, Burlingame, CA, USA) according to the manufacturer’s instructions. Purple stain was used to avoid confusion with brown melanin pigment, and methyl green (H-3402, Vector^®^ Labs, Burlingame, CA, USA) was used to counterstain. Human liver, placenta, and kidney obtained from autopsy were used as positive controls. Secondary antibody without primary antibody was used as a negative control. All samples were evaluated as positive or negative for cytoplasmic or nuclear staining. Slides were digitalized with Panoramic Scan instrument and examined with Panoramic Viewer software (3DHISTECH Ltd., Budapest, Hungary). According to the intensity, the proportion of positive cells was ranked in five groups: 0 (negative), 1 (1–25%), 2 (26–50%), 3 (51–75%) and 4 (76–100%). As an extra positive control, placental tissue was used for VEGFA staining, and normal kidney tissue was used for HIF1A staining. The content of melanin pigments of the specimens was also evaluated in all 52 samples. Stained tumor sections were reviewed by two independent observers, masked to patient outcome and variables.

### 2.6. Statistical Analysis

Statistical associations between FZD6, VEGFA and HIF-1α gene expression of the paraffin-embedded tissues were evaluated with Spearman correlation analysis. Survival of the patients depending of the variables was plotted against the post-operative days (elapsed until death or the follow-up period) according to the Kaplan–Meier method. Calculations were performed using IBM SPSS Statistics (IBM Corp. Released 2014. IBM SPSS Statistics for Windows, Version 23.0. Armonk, NY, USA: IBM Corp.). Results of the Western blot analysis was evaluated using the Prism 5 software (GraphPad Software, Inc., San Diego, CA, USA). Data are presented as a mean value ± standard error of the mean (SEM).

## 3. Results

### 3.1. Investigation of the Gene Expression of Nestin, NGFR, SOX10, FZD6 and PROM1 Cancer Stem Cell Markers in 18 Human UM Specimens and in Three Normal Uvea Samples

Snap frozen uveal melanoma tissue specimens consisted of seven epitheloid, eight spindle and three mixed cell type tumors from 6 female and 12 male patients. Clinical data are summarized in Table 2.

**Figure 1:**
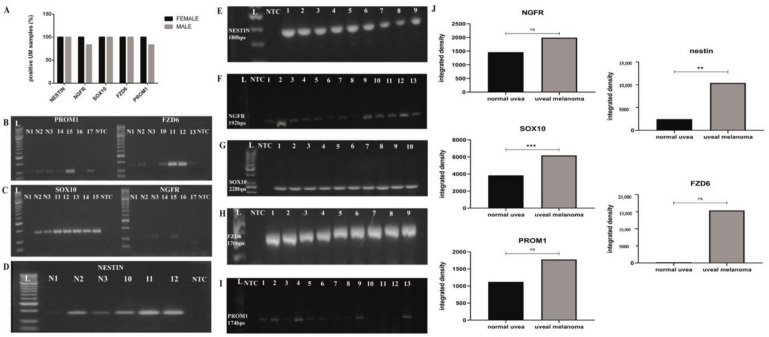
Representative RT-PCR analysis of mRNA expression of stem cell markers in normal uvea and uveal melanoma specimens. (**A**) Gender distribution of investigated stem cell markers in human UM samples; (**B**–**D**) Normal uvea specimens showed a lower positivity for stem cell markers compared to the enucleated UM samples; L: 50 bps DNA ladder, NTC: no template control, N1–N3: normal uvea specimens, No.1–18: representative human UM samples; (**E**–**I**): Representative agarose gel electrophoresis photos with the expression of nestin, NGFR, SOX10, FZD6 and PROM1 stem cell markers in representative human UM samples (No.:1–18.); L: DNA ladder: 50 bps ladder, NTC: no template control; (**J**) densitometric analysis of the expression of NGFR, SOX10, PROM1, nestin and FZD6 cancer stem cell markers in three normal uvea and 18 UM samples (**: highly significant, *p* < 0.01; ***: extremely significant, *p* < 0.005.

The expression of cancer stem cell markers in 18 human UM and in three normal uvea specimens was demonstrated by RT-PCR (Figure 1). Densitometric analysis of the agarose gel electrophoresis of the PCR products from normal uvea samples showed considerably lower positivity compared to tumor samples (nestin: *p* = 0.007; SOX10: *p* = 0.004). The lower expression of the five stem cell markers could be explained by the limited number of stem cells in healthy tissues. Normal uvea samples showed markedly lower positivity in the densitometric analysis based on the agarose gel electrophoresis of the PCR products (nestin: *p* = 0.007; SOX10: *p* = 0.004). The lower expression of the five stem cell markers can be explained by the limited number of stem cells in healthy tissues. The 18 UM specimens showed stronger stem cell marker expression with the exception of NGFR. mRNA levels of nestin, FZD6 and SOX10 could be detected in all the samples. PROM1 was expressed in 82% and *NGFR* was expressed in 94% of the specimens. We further analyzed the results of five patients previously treated with ruthenium applicator and we found that all of them expressed the five stem cell markers investigated similarly to the UM specimens not treated with ruthenium. No effect of previous ruthenium therapy on the expression of stem cell markers was detected in our study.

### 3.2. Protein Expression of FZD6, HIF-1α and VEGFA Genes in 18 Human Snap Frozen UM Specimens

**Figure 2:**
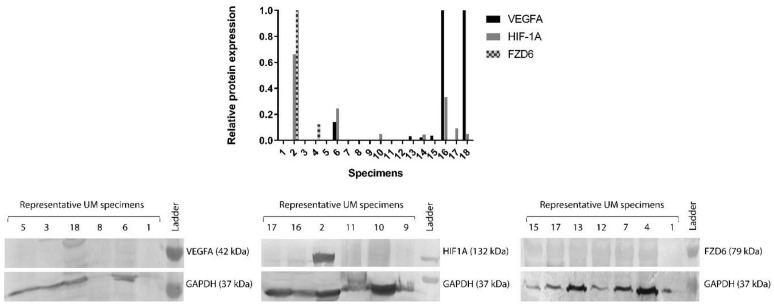
Protein expression of VEGFA, HIF-1α and FZD6 genes in 18 uveal melanoma specimens. Of the samples, 11.11% were positive for FZD6, 38.88% and 33.33% of the samples were positive for the expression of HIF-1α and VEGFA, respectively. Data represent the densitometric analysis of the target genes normalized to GAPDH.

To confirm the RT-PCR results, protein levels of FZD6, HIF-1α and VEGFA have also been examined with SDS-PAGE-Western blot analysis. We investigated protein samples of 18 UM patients, that were previously examined by RT-PCR. Our results showed that only a low number of samples (11.11%) were positive for FZD6; 38.88% and 33.33% of the samples were positive for the expression of HIF-1α and VEGFA, respectively (Figure 2).

### 3.3. Clinical, Pathological, and Molecular Biomarkers of UM Specimens in Tissue Microarray

Considering the low number of samples, we decided to modify our method in order to be able to investigate a great number of UM specimens with a more sensitive and cost-effective tissue microarray (TMA) system. Our study included 22 women and 30 men, with an average age of 59.55 years (range between 30 and 83 years). The paraffin-embedded primary uveal melanoma specimens consisted of 16 epitheloid, 24 spindles and 12 mixed cell type tumors. Only three patients were diagnosed with distant metastasis. Clinical data are summarized in Table 3.

The results of the immunostaining were suitable for the evaluation for FZD6 in 52 cases, for HIF-1α in 50 cases for VEGFA and in 48 cases.

### 3.4. Overall Survival Could Depend on the Tumor Cell Type Constituting the UM Tumor

**Figure 3:**
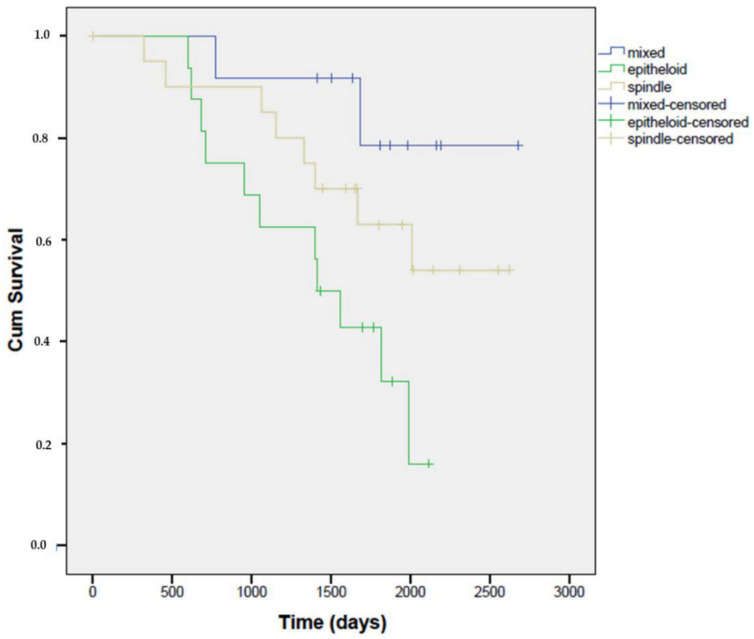
Kaplan–Meier curves of survival in patients with primary uveal melanoma related to tumor cell type. The best survival rate was associated with the proportion of patients with mixed cell type. The worst survival rate was observed with the epitheloid subtype (Mantel–Cox test, n = 49, *p* = 0.02). Kaplan–Meier curves are plotted against the number of postoperative days depending on the tumor cell type.

Kaplan–Meier curves showed a significant difference in overall survival depending on the tumor cell type. These results are in agreement with previous findings, namely that the histotype of uveal melanoma has an influence on the survival of the patient. Our results confirmed that the epithelioid subtype is associated with a worse prognosis than the other two tumor subtypes. The best survival rate was associated with the proportion of patients with mixed cell type, the worst survival rate was observed with the epitheloid subtype (Mantel–Cox test, n = 49, *p* = 0.02) (Figure 3).

### 3.5. FZD6 Expression Is Related to VEGFA Expression and Has an Influence on the Survival Rate of the Patients with Primary UM

Positive immunostaining for FZD6 was found in 52 samples with different intensity of expression. In 28 cases (58.8%) we detected mild positivity (1+), in seven cases (13.7%) intense positivity (2+) and 17 cases (29%) no expression was detected. Normal nevi samples were negative to FZD6 expression. According to the Spearman statistical analysis, there is a statistically significant strong correlation (Spearman r = 0.411, n = 48, *p* = 0.004) between FZD6 and VEGFA expression, but not between FZD6 and HIF-1α expression (Spearman r = 0.061, n = 50, *p* = 0.672).

**Figure 4:**
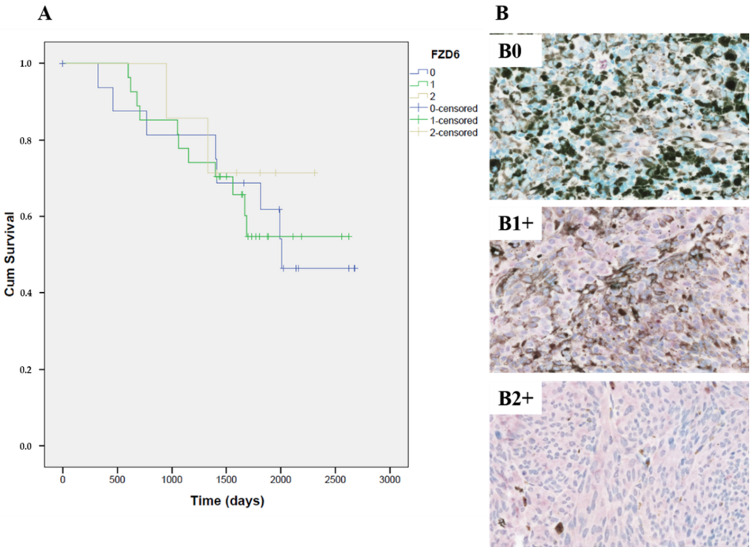
Expression of FZD6 stem cell marker in enucleated human uveal melanoma specimens. (**A**) Kaplan–Meier curves of survival in patients with primary uveal melanoma related to the intensity of FZD6 stem cell marker. There is no significant correlation (Mantel–Cox test, n = 51, *p* = 0.867) between the FZD6 expression and the survival of the patients; (**B**) Images of the representative samples immunostained for FZD6, B0: tumor sample without detectable (0) FZD6 expression, B1+: tumor sample with mild positivity (1+) for FZD6 in the cytoplasm of the tumor cells, B2+: tumor sample exhibits intense expression (2+) for FZD6 in the cytoplasm of the tumor cells. Original magnifications of all images: 400×. Images B0, B1+ and B2+ are peroxidase substrate V-VIP stained sections with methyl green counterstaining.

Kaplan–Meier curves showed no significant correlation (Mantel–Cox test, n = 51, *p* = 0.867) between the FZD6 expression and the survival of the patients, however, the slope of the Kaplan–Meier curves of samples with 0 and 1+ positivity noticeably indicates a poor prognosis (Figure 4).

**Figure 5:**
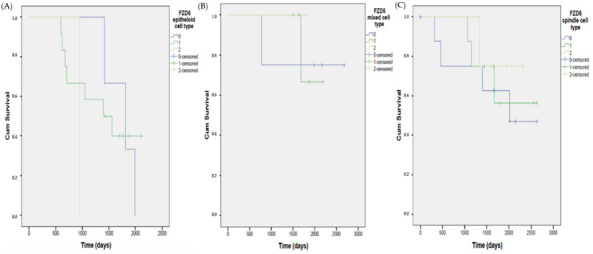
Kaplan–Meier curves show different overall survival rates for uveal melanoma patients with FZD6 expression according to tumor cell type. Based on the Kaplan–Meier curves there is a clear tendency for poor survival rate but no significant correlation is revealed (Mantel–Cox test, n = 16, *p* = 0.541, epitheloid subtype) between the three tumor subtype (**A**)–(**C**) of UM samples.

Comparing the survival rate of the three tumor subtypes separately and considering their FZD6 expression levels, an obvious but not significant difference was revealed. Based on the Kaplan–Meier curves (plotted against the number of postoperative days depending on FZD6 expression) there was a detectable trend (Mantel–Cox test, n = 16, *p* = 0.541) for a poor survival rate in the epithelioid subtype compared to the other two subtypes of UM (Figure 5.).

### 3.6. No Significant Correlation Was Detected between the Expression of Angiogenic Factors and Survival Rates in Primary UM Specimens

Positive immunostaining for VEGFA was found in 48 samples with different intensity of expression. In five cases (10.4%) we detected strong positivity (4+), in 12 cases (25%) high positivity (3+), in 12 cases (25%) intense positivity (2+), in 12 cases (25%) mild positivity (1+) and in seven cases (14.6%) no expression was detected. Normal nevi samples were negative to FZD6 expression.

Survival analysis showed no significant correlation between the VEGFA expression and the survival rate (Mantel–Cox test, n = 47, *p* = 0.757) (Supplementary Figure S1).

FZD6 and VEGF expression had no significant effect on survival, despite their correlation confirmed by the Spearman test and the considerable similarity between their survival curves.

Positive immunostaining for HIF-1α was found in 50 samples with different intensity of expression. In 10 cases (20%) we detected strong positivity (4+), in 26 cases (52%) high positivity (3+), in 10 cases (20%) intense positivity (2+), in two cases (4%) mild positivity (1+) and in two cases (4%) no expression was detected. Normal nevi samples showed high positivity (3+) to HIF-1α expression.

No correlation was found between the HIF-1α expression and the survival of the patients (Mantel–Cox test, n = 49, *p* = 0.336) (Supplementary Figure S2).

### 3.7. The Expression of Melanin Pigment Negatively Correlates with the Overall Survival Rate in Patients with Primary UM

**Figure 6:**
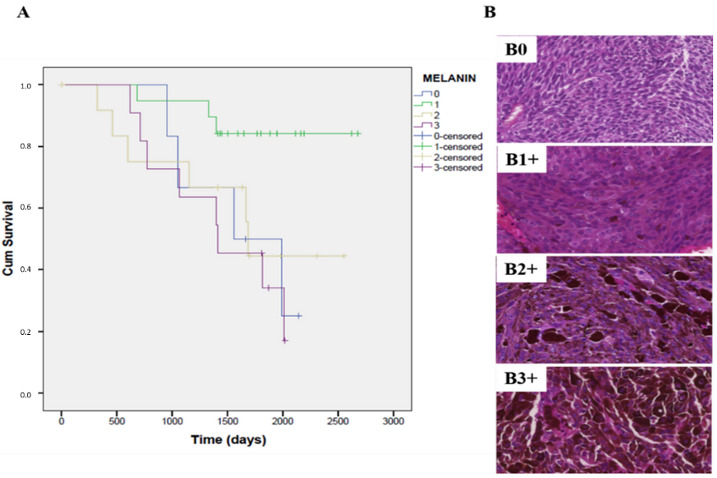
Survival rate association of enucleated human uveal melanoma specimens based on melanin pigment content. (**A**) Kaplan–Meier curves estimates of the overall survival related to melanin content of 52 uveal melanoma tumors (Mantel–Cox test, n = 49, *p* = 0.033). The highest survival rate was associated with the samples in which the proportion of positive cells was ranked to quartile 1 (1–25%); (**B**) Methyl green counterstained sections of a representative sample showing melanin-producing neoplastic human uveal melanoma cells; (B0) Tumor sample without melanin pigment expression (0) in the cytoplasm of the tumor cells; (B1+) Tumor sample exhibits mild positivity (1+) for melanin in the tumor cell; (B2+) intense expression (2+) for melanin in the cytoplasm of the tumor cells; (B3+) high expression (3+) for melanin in the cytoplasm of the tumor cells. Original magnifications of all images: 400×.

In 10 cases (20.4%) we detected high positivity (3+), in 13 cases (26.53%) intense positivity (2+), in 20 cases (40.81%) mild positivity (1+) and in six cases (12.24%) no expression of melanin was detected. Kaplan–Meier curves showed a significant difference in the overall survival depending on the melanin content of the samples (Mantel–Cox test, n = 49, *p* = 0.033). The highest survival rate was associated with the samples in which the proportion of positive cells was ranked to the lowest quartile (Q1:1–25%) where the expression of melanin was only weak (Figure 6).

Furthermore, we compared the results of the TMA analysis with other pathological markers, such as sclera and nervus opticus infiltration but no statistically significant association was found. (Supplementary Table S1).

## 4. Discussion

A growing body of evidence shows that tumorigenic cancer stem cells exist in human melanomas [31,34,35,38]. Human uveal melanoma is composed of distinct cell types reminiscent of neural crest derivatives and UM contains multipotent cells that express the neural crest stem cell markers [31,34,40,41] Chang *et.al.* has reported that class 2 UM (higher risk to metastasis) is strongly associated with genes upregulated in primitive neuroectodermal cells the phenotype of which is related to increased vascular density [29,31,34,41].

The cancer stem cell hypothesis has been confirmed in human malignant melanoma tumors. Many publications showed that cells functioning with special markers and signal transduction pathways which are typical of pluripotent stem cells are responsible for the initiation, progression and drug resistance of melanoma [29,32,38,39,42]. Previous studies have described that the population of vascular mimicry-forming uveal melanoma cells can acquire a cancer stem cell-like phenotype that may play a role in the increased therapy resistance of these cells or in the metastatic spread of the tumor [43,44]. Moreover, other studies have described the presence of CSC-like subpopulations in UM cell lines, with enhanced proliferative capabilities [30,31,34,41].

The hypothesis of our recent study was that central regulators of angiogenesis and metastasis could be influenced by CSCs to enhance the ability of the tumor to develop a chemoresistant phenotype. Since the previously published data on stem cells and neuro stem cell-like phenotype showed a higher risk for metastasis, we wanted to investigate central factors supporting the successful spread of the tumor [29,36,44,45,46]. Furthermore, we aimed in the present study to evaluate the expression of nestin, NGFR, Sox10, FZD6 and PROM1 cancer stem cell markers and angiogenic factors in UM and to better understand the relationships between the detected markers and vasculogenic mimicry patterns.

Our results from the investigation of 18 primary UM specimens indicate a higher amount of primitive neuroectodermal stem cells compared to normal uvea samples. Notably, 100% of the samples were positive for nestin, FZD6 and SOX10; 82% for PROM1 and 94% NGFR in the UM tumors. Based on the special characteristics of uveal melanoma, namely, that it can spread only hematogenously, our aim was to further investigate the correlation between FZD6 and the genes related to angiogenesis and migration in UM. FZD6 (frizzled class receptor 6) is a 7-transmembrane domain protein that are receptors for non-canonical Wnt signaling proteins that play an important role in cell division, cell adhesion, migration and also plays a crucial role in human tumorigenesis [45,46,47,48]. Although the biomarkers of CSCs in UM have not been well established, it is known, that the Wnt/β-catenin pathway is a key intrinsic regulator of self-renewal of CSCs [46]. Previous studies showed that Tenovin-6 is effective to induce apoptosis in UM cells and eliminate CSCs by suppressing the expression of β-catenin [49].

In order to investigate the associations between FZD6 and the angiogenic factor HIF-1α and VEGFA, we performed Western blot analysis using 18 UM specimens. Unfortunately, only 11.11%, 38.88% and 33.33% of the samples showed representative and positive results for FZD6, HIF-1α and VEGFA.

In our subsequent experiments, we investigated the expression of FZD6, VEGFA and HIF1A genes in 52 paraffin-embedded primary UM samples with the tissue microarray (TMA) technique. Based on the results, we found correlations between angiogenic mimicry, the presence of CSCs and overall survival.

The main purpose of this study was to evaluate the statistical relationships and relative significance between FZD6 and angiogenic biomarkers, e.g., HIF-1α and VEGFA to employ bioinformatic tools in order to explore potential biological explanations for the co-occurrence of these biomarkers in a highly metastatic and chemoresistant tumor. Spearman analysis showed a statistically significant (*p* = 0.004) and strong correlation between FZD6 and VEGFA expression. This association between these biomarkers suggest a presence of special signals between these biomarkers in UM tumors, with the goal of vascularization and spread of the primary tumor. Unfortunately, Kaplan–Meier curves showed no significant correlation between the FZD6 or VEGFA expression and the survival of the patients. It can be explained by the relatively low amount of samples, especially in the case of curves plotted against subtypes of the tumors. Surprisingly, survival curves showed no correlation between HIF-1α expression and FZD6 or VEGFA expression, despite targeting VEGFA, often be assumed to be a promising way to treat a densely vascularized UM tumor. Furthermore, we detected an interesting negative correlation between the pigmentation of the tumor and the survival of the patient. Kaplan–Meier curves showed a significant difference in overall survival depending on the melanin content of the samples (*p* = 0.033). Based on these results, we suggest a worse prognosis for the patients with considerable amounts of melanin (ranked in Q2: (26–50%), Q3: (51–75%)).

The biological significance of stem cell markers, such as FZD6 in primary uveal melanomas still remains unclear. How could such a stem cell-like expression profile explain the tendency towards vascularization in UM tumors? Perhaps UM cancer cells with a stem cell-like phenotype have a survival advantage during hematogenous transit and colonization of metastatic sites. We suggest that this can be the background of the detected correlation between FZD6 and VEGFA expression. Although our findings are preliminary and observational in nature, they strongly suggest that further experimental work is needed to explore the role of cancer stem cells in uveal melanoma and the implications for clinical management and novel therapies.

## Figures and Tables

**Table 1:** The primer pairs used in RT-PCR reactions to investigate the presence of cancer stem cells in human normal and uveal melanoma tissue specimens.

Primers	Sequence	PCR Product Size
Nestin	Forward: 5′-AGAACTCCCGGCTGCAAA-3′	180 bps
	Reverse: 5′-GCACAGGTGTCTCAAGGGTAG-3′	
NGFR	Forward: 5′-TGCCAGGACAAGCAGAAC-3′	192 bps
	Reverse: 5′-GGGTGTGGACCGTGTAATC-3′	
SOX10	Forward: 5′-CGTCAGCCAGGTGCTCAG-3′	228 bps
	Reverse: 5′-CGCTTGTCACTTTCGTTCAG-3′	
PROM1	Forward: 5′-GCACTTACGGCACTCTTCAC-3′	174 bps
	Reverse: 5′-TTCCACAAGCAGCAAAATCC-3′	
FZD6	Forward: 5′-TGAGCAAGTGAACAGGATTACC-3′	170 bps
	Reverse: 5′-CCCAGAAGACAGCAGAGATG-3′	
β-actin	Forward: 5′-GGCATCCTCACCCTGAAGTA-3′	200 bps
	Reverse: 5′-GGGGTGTTGAAGGTCTCAAA-3′	

**Table 2:** Clinical and pathological features of tissue specimens from 18 patients with uveal melanoma.

Clinical and Pathological Features	n = 18
Average age (range)	63.1 (30–84)
Female (patient)	6
Male (patient)	12
Epitheloid tumor cell type (patient)	7
Spindle tumor cell type (patient)	8
Mixed tumor cell type (patient)	3
Median tumor diameter (range)	9.22 mm (2.5–23)
Median tumor thickness (range)	7.19 mm (1–16)
Patients with sclera infiltration	9
Patients without sclera infiltration	9
Patients with nervus opticus infiltration	2
Patients without nervus opticus infiltration	16
Median follow-up time (years, range)	3.71 (1.70–6.37)
Ruthenium applicator (patient)	5

**Table 3:** Clinical and pathological features of tissue specimens in tissue microarray from 52 patients with uveal melanoma.

Clinical and Pathological Features	n = 52
Average age (range)	59.55 (35–83)
Female (patient nr.)	22
Male (patient nr.)	30
Epitheloid tumor cell type (patient nr.)	16
Spindle tumor cell type (patient nr.)	24
Mixed tumor cell type (patient nr.)	12
Median tumor diameter (range)	12.68 mm (2.5–23)
Median tumor width (range)	9.56 mm (5–14)
Median tumor thickness (range)	6.47 mm (1–16)
Patients with sclera infiltration	26
Patients without sclera infiltration	26
Patients with nervus opticus infiltration	3
Patients without nervus opticus infiltration	49
Median follow-up time (years, range)	4.43 (1.1–7.33)
Ruthenium applicator (patient)	3

## Supplementary Materials

The following are available online at https://www.mdpi.com/2075-1729/10/12/310/s1, Figure S1: Kaplan–Meier curves showed no significant correlation between the VEGFA expression and the survival rate (Mantel–Cox test, n = 47, *p* = 0.757), Figure S2: No correlation was found between the HIF-1α expression and the survival of the patients with UM (Mantel–Cox test, n = 49, *p* = 0.336), Table S1: Spearman statistical analysis showed no significant association between pathological markers, such as sclera and nervus opticus infiltration and the expression of genes investigated in this study.
