# Supplementary material for: Correlation between the Expression of Angiogenic Factors and Stem Cell Markers in Human Uveal Melanoma
Source: Life (Basel). 2020 Nov 25;10(12):310. doi: 10.3390/life10120310 (PMC7760175; doi:10.3390/life10120310)
Supplement: Supplementary file 1 [file life-10-00310-s001.pdf]

life

MDPI

Supplementary Materials

Correlation between the Expression of Angiogenic Factors and Stem Cell Markers in Human Uveal Melanoma

Table S1. Spearman statistical analysis showed no significant association between pathological markers, such as sclera and nervus opticus infiltration and the expression of genes investigated in this study.

		FZD6	HIF1α	VEGFA	Melanin
Sclera infiltration	Correlation coefficient	-,200	319*	,035	-,020
	Sig. (2-tailed)	,198	,042	,830	,902
	N	43	41	39	41
Nervus opticus infiltration	Correlation coefficient	-,192	,139	-,007	-,126
	Sig. (2-tailed)	,192	,358	,962	,406
	N	48	46	44	46

*Correlation is significant at the 0.05 level (2-tailed probe).

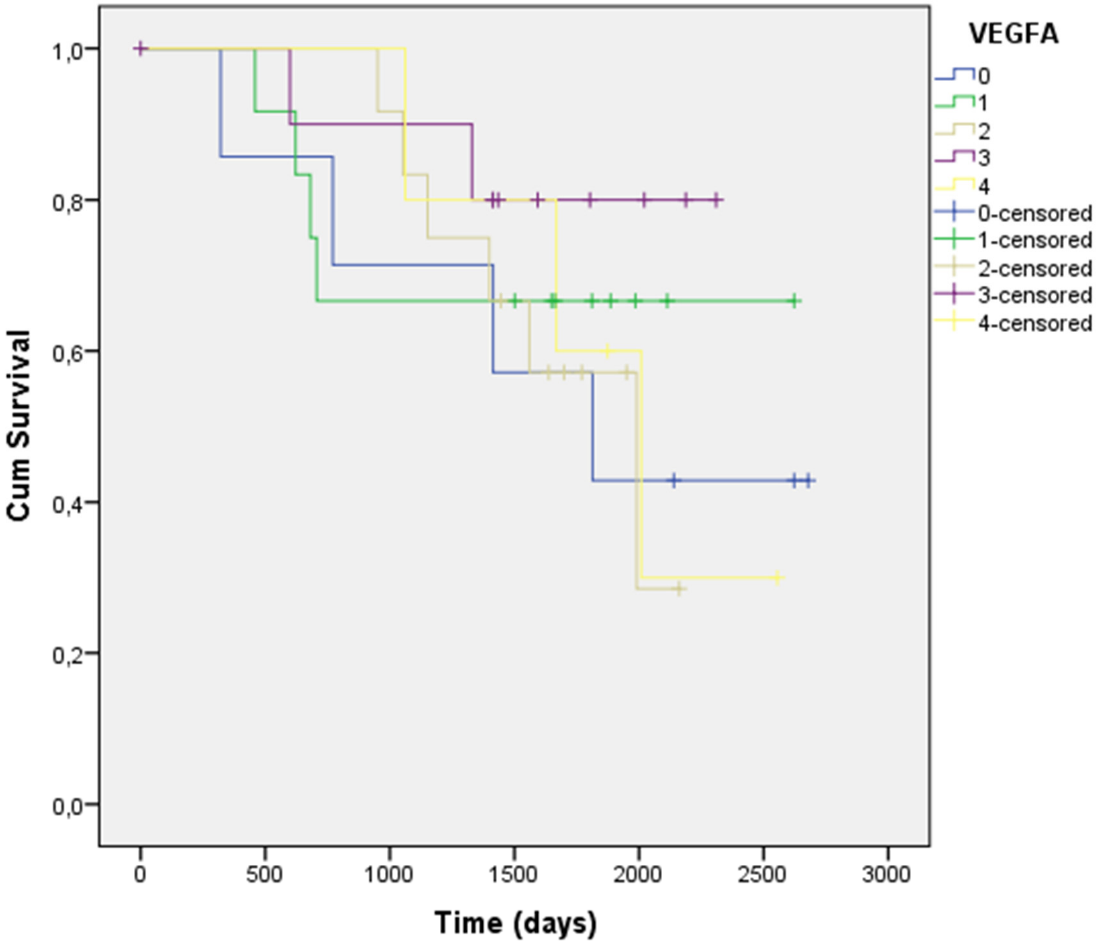

Figure S1. Kaplan–Meier curves showed no significant correlation between the VEGFA expression and the survival rate (Mantel–Cox test, n = 47, p = 0.757).

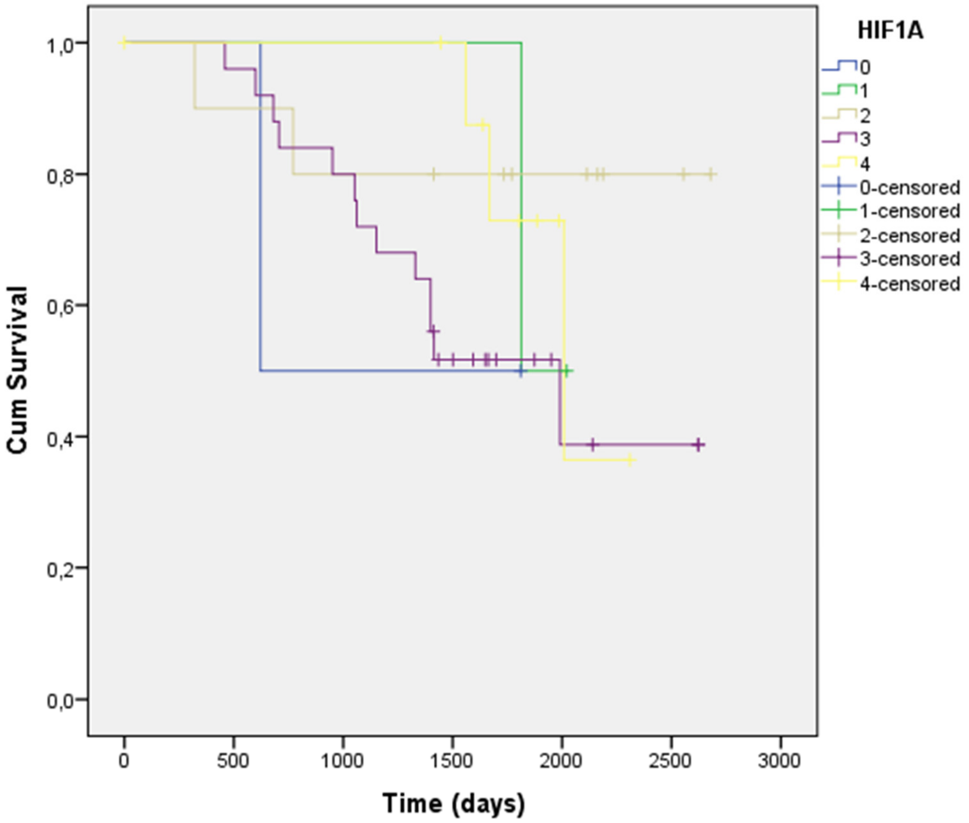

Figure S2. No correlation was found between the HIF-1α expression and the survival of the patients with UM (Mantel–Cox test, n = 49, p = 0.336).

Publisher's Note: MDPI stays neutral with regard to jurisdictional claims in published maps and institutional affiliations.

© 2020 by the authors. Licensee MDPI, Basel, Switzerland. This article is an open access article distributed under the terms and conditions of the Creative Commons Attribution (CC BY) license (http://creativecommons.org/licenses/by/4.0/).
